# Preparation of Fe_3_O_4_/Chitosan–Acrylic Acid Nanocomposite as an Adsorbent for the Removal of Cu^2+^ Ions From Real Water Samples

**DOI:** 10.1049/nbt2/9919353

**Published:** 2025-11-23

**Authors:** Arman Samadzadeh Mamaghani, Ali Moghimi, Milad Abniki

**Affiliations:** ^1^Department of Chemistry, South Tehran Branch, Islamic Azad University, Tehran, Iran; ^2^Department of Pharmaceutical Chemistry, TeMS.C., Islamic Azad University, Tehran, Iran; ^3^Department of Resin and Additives, Institute for Color Science and Technology, Tehran, Iran

**Keywords:** adsorption, copper ions, magnetic sorbent, magnetic/chitosan–acrylic acid adsorbent, removal

## Abstract

This study presents an environmentally friendly and nontoxic method for the selective separation and removal of trace amounts. A magnetic nanocomposite made of Fe_3_O_4_/chitosan–acrylic acid was utilized to separate and remove Cu^2+^ ions using its magnetic properties. Various characterization techniques, including Fourier transform infrared (FTIR) spectroscopy, X-ray diffraction (XRD), scanning electron microscope (SEM), thermogravimetric analyzer (TGA), and VSM, were employed to investigate and identify the nanocomposite. Additionally, the research discusses adsorption isotherm models related to the adsorption of Cu^2+^ ions. The maximum adsorption capacity of the biodegradable Fe_3_O_4_/chitosan–acrylic acid nanocomposite for Cu^2+^ ions was found to be 30.68 mg/g. The adsorption process followed the Freundlich isotherm model when using the Fe_3_O_4_/chitosan–acrylic acid adsorbent. The method exhibited a linear range of 10–1000 µg/L for Cu^2+^ ions, with a limit of detection (LOD) of 0.15 μg/L for the adsorption of Cu^2+^ ions by the Fe_3_O_4_/chitosan–acrylic acid adsorbent. These findings indicate that Fe_3_O_4_/chitosan–acrylic is a high-performance adsorbent for removing Cu^2+^ ions from tap, well, river, and spring water samples.

## 1. Introduction

The presence of heavy metal ions, such as Cu(II) ions, in water poses a significant threat to both human health and the ecosystem. Sources of Cu(II) ions' contamination include industrial effluents, agricultural runoff, and certain household products. To address these harmful effects, it is essential to develop effective and efficient methods for removing Cu(II) ions from water samples. Recent studies have investigated various techniques for the removal of copper (II) ions, including adsorption, ion exchange, membrane filtration, and electrochemical methods. Factors such as selectivity, capacity, cost-effectiveness, and environmental impact have also been evaluated to assess the advantages and limitations of each approach [1–3].

The threat posed to human health underscores the urgent need for a fast, sensitive, and reliable analytical method to measure heavy metals in environmental waters and industrial effluents. However, these methods can be labor-intensive and often result in the analytes coming into contact with organic solvents, which may alter their composition due to the influence of the solvent. Additionally, the excessive use of solvents generates waste that can enter the environment, raising concerns about their negative impact [4]. Another common method for sample preparation is solid-phase extraction, which uses less solvent than the previously mentioned methods. As a result, the analyte remains relatively intact, and less waste is produced.

Dispersive solid-phase extraction is a specific type of solid-phase extraction in which a solid adsorbent is dispersed throughout the sample solution. After the adsorption process, the adsorbent is reused after washing it with a suitable solvent [5, 6]. This method increases the contact surface area compared to traditional solid-phase extraction techniques, where the solid-phase is typically arranged in the form of a column or disk. Furthermore, the incorporation of magnetic properties into the adsorbent has simplified its separation from the sample solution, resulting in the development of a new method known as magnetic solid-phase extraction (MSPE) [7, 8].

There are several methods used to remove heavy metals from industrial wastewater, including ion exchange, solvent extraction, chemical precipitation, adsorption, nanofiltration, biological sequestration, and reverse osmosis [9, 10]. Among these methods, adsorption is the most commonly used for copper removal due to its cost-effectiveness, minimal waste formation, high efficiency, ease of operation, and effective removal rates [2]. Effective adsorbents for removing heavy metals from water include carbon-based adsorbents, organic adsorbents, natural adsorbents, inorganic nanoparticles, and organic frameworks [11–17].

Chitosan is a highly effective adsorbent, boasting a superior adsorption rate compared to other types of adsorbents for the removal of Cu^2+^ ions. This biodegradable and biocompatible adsorbent, due to its large specific surface area, with a large number of hydroxyl, amino, and acetamido groups, has been widely used for the removal of inorganic and organic pollutants from the aqueous environment [18].

Magnetic nanocomposites are an advanced group of materials that integrate nanoscale magnetic elements with diverse matrices, such as polymers, ceramics, or carbon-based frameworks, to exhibit exceptional physical, chemical, and magnetic properties. Recent advances in synthesis and materials engineering methods have led to innovative magnetic nanocomposites with superior performance that are suitable for applications in biomedicine, energy storage, environmental remediation, and advanced magnetic technologies. New magnetic nanocomposites are reviewed, focusing on their synthesis techniques, structural features, and emerging applications, emphasizing their potential to address global challenges. In this paper, the properties of a chitosan-based nanocomposite as a natural polymer and an acrylic acid-derived polymer as a synthetic polymer in the presence of iron oxide nanoparticles for the removal of copper ions in aqueous media are investigated [19].

By using cross-linking and grafting an active agent onto chitosan, its stability and adsorption capacity have been increased [3, 20–22]. For example, Al-Karawi et al. [23] modified the surface of chitosan with acrylamide to increase the binding site and adsorption capacity for Cu^2+^ ions. In this study, the researchers found that, at pH 7 and a temperature of 60°C, the highest adsorption of copper metal by the acrylamide-grafted chitosan adsorbent occurred, and the reaction reached equilibrium in a time between 12 and 18 h [24].

In this study, we aim to develop a new type of modified magnetic nanocomposite by chitosan–acrylic acid. This biocompatible compound is designed for the efficient removal of Cu(II) ions. After characterizing the biocompatible nanoadsorbent, we optimized several parameters affecting its adsorption efficiency. We studied the adsorption kinetics and isotherms by fitting the experimental data to different models, and we proposed a mechanism for the adsorption process. Additionally, we investigated the thermodynamics of adsorption. The results indicated that the proposed adsorbent demonstrates higher adsorption efficiency and adsorption capacity for removing Cu(II) from environmental water samples compared to other developed solid-phase extraction adsorbents.

## 2. Materials and Methods

### 2.1. Materials

Acrylic acid (98.0%), N, N′-methylenebisacrylamide (97.0%), and chitosan (deacetylation degree of 96%) were procured from Sigma–Aldrich (USA). Ammonium persulfate (98%), FeCl_3_·6H_2_O (99%), FeCl_2_.4H_2_O (99%), Cu(NO_3_)_2_·3H_2_O (98%), Mg(NO_3_)_2_·6H_2_O (98%), Ca(NO_3_)_2_·4H_2_O (99%), NaNO_3_ (≥ 99%), and KNO_3_ (≥ 99.0%) were obtained from Merck (Darmstadt, Germany). A stock solution of Cu^2+^ was prepared by dissolving Cu(NO_3_)_2_·3H_2_O in deionized water to achieve a concentration of 300 mg/L. Throughout the study, analytical-grade chemicals were employed, and deionized water was used in all experiments.

### 2.2. Characterizations

A thermogravimetric analyzer (TGA2, Mettler Toledo, Switzerland) was used to assess the heat stability of the hydrogel. Additionally, Fourier transform infrared (FTIR) spectroscopy was performed using a spectrometer from Perkin Elmer (USA). The adsorption of copper ion concentrations was tested with a graphite furnace atomic spectrometer (Agilent, USA). X-ray diffraction (XRD) patterns were analyzed using X'Pert PRO diffractometer (Panalytical, Netherlands). The specific surface area of the Fe_3_O_4_/chitosan–acrylic acid nanocomposite was determined using a multiple-point BET method with a BELSORP MINI II (BEL, Japan). Photos were captured using a field emission scanning electron microscope (FESEM, ZEISS Sigma VP, Germany). Magnetization measurements were conducted with a VSM AGFM/VSM3886 device from Meghnatis Daghigh Kavir Company (Iran). Finally, magnetic separation was performed using a super magnet that produced a magnetic field of 1.2 T (N35 model from Tehran Magnet, Tehran, Iran).

### 2.3. Synthesis of Nano Magnetic Iron Oxide

To begin, 2.5 g of FeCl_2_·4H_2_O and 5 g of FeCl_3_·6H_2_O were each dissolved in 50 mL of distilled water. These two solutions were then combined in a flask. The mixture was stirred at high speed for 1 h at 60°C and continuously exposed to nitrogen gas, while 50 mL of 3 M sodium hydroxide (NaOH) was slowly added to the mixture. Afterward, the mixture was rinsed with distilled water to adjust the pH level to 7, and finally, it was dried in an oven at 70°C for 24 h.

### 2.4. Magnetic Nanocomposite Preparation

**Figure 1:**
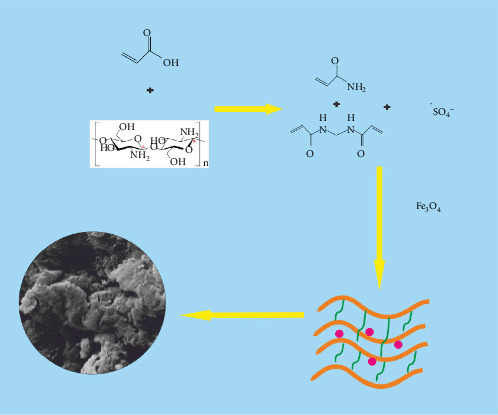
Synthesis of the Fe_3_O_4_/chitosan–acrylic acid nanocomposite.

To prepare the hydrogel, 5 mL of acrylic acid monomer was dissolved in 20 mL of distilled water. Next, 0.1 g of chitosan was added to the solution. The mixture was stirred well for 4 h before filtering it. In a separate step, 5 mL of acrylic acid was dissolved in 20 mL of distilled water. Then, the acrylic acid/chitosan filtered solution was combined with 0.10 g of nano iron oxide that had been dissolved in 10 mL of water in a small beaker. The mixture was stirred using a glass stirrer. Afterward, 0.20 g of ammonium persulfate and 0.05 g of N, N′-methylenebisacrylamide were added to the beaker. The beaker was placed in a temperature-controlled environment at 70°C under a nitrogen atmosphere to facilitate polymerization for 2 h. Once the polymerization process was complete, the polymer was dried in a vacuum oven at a temperature of 60°C (as illustrated in Figure 1) [3].

### 2.5. Sorption Experiments

This study focused on Cu^2+^ ions to conduct a series of adsorption experiments aimed at testing the efficacy of hydrogel materials for contaminant removal. The concentration of adsorbed copper ions was measured using a graphite furnace atomic spectrometer. A 1 g/L Cu^2+^ stock solution was prepared for use in the adsorption experiments. To quantify the adsorption capacity (qe) of Cu^2+^ ions (mg/g), Equation (1) was utilized as referenced in [25, 26].

(1) $$\begin{array}{c} \text{qe}=\frac{\left(C0-\text{Ce}\right)V}{m}. \end{array}$$

In this experiment, the volume of the solution (*V*) is measured in mL, and the (m) weight of the adsorbent (mg) and the initial (*C*_0_) and final (*C*_e_) concentrations of Cu^2+^ ions are measured (mg/L). The pH of the Cu^2+^ ions solution was adjusted to a range between 3.0 and 9.0 using either 1.0 M hydrochloric acid (HCl) or 1 M NaOH to assess the effect of pH on adsorption effectiveness. Additionally, ammonia and ammonium buffer solutions were utilized for experiments conducted at alkaline pH levels. All adsorption experiments were performed in triplicate, and the average values were reported to ensure data reliability [24, 27, 28].

## 3. Results and Discussion

### 3.1. Characterizations

#### 3.1.1. FTIR Analysis

**Figure 2:**
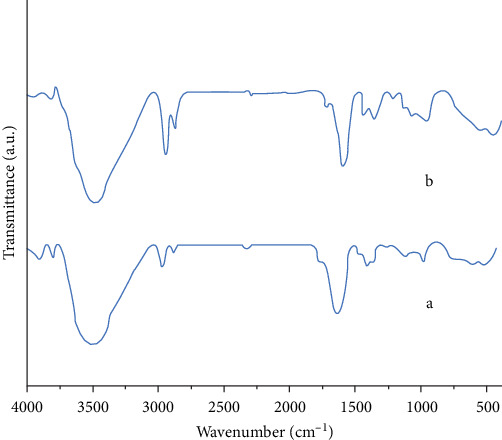
FTIR spectra of the Fe_3_O_4_/chitosan–acrylic acid nanocomposite: (a) before Cu^2+^ ions adsorption and (b) after Cu^2+^ ions adsorption.

Figure 2 presents the findings from the FTIR investigation of the magnetic chitosan hydrogel adsorbent (Figure 2a). The peak at 3442 cm^−1^ in the FTIR spectra corresponds to the hydroxyl (─OH) groups. The ─CH stretching bonds are represented by the peaks at 2928 cm^−1^ and 2858 cm^−1^ [29, 30]. Additionally, the stretching vibration of the carbonyl (C═O) group peaks at 1627 cm^−1^. The bending vibration associated with the Fe─O bond in Fe_3_O_4_ is indicated by the peak at 777 cm^−1^ [31]. The peak at 1458 cm^−1^, related to the N─H group, demonstrates the link between the polyacrylamide and the amine groups of chitosan [3, 20–25, 27–35]. The FTIR spectra after the adsorption of Cu^2+^ ions are shown in Figure 2b. Upon the addition of Cu^2+^ ions, the broad peak of the adsorbent attributed to NH or OH at 3445 cm^−1^ shifted to 3446 cm^−1^ and 3782 cm^−1^, indicating that the NH or OH groups may interact with Cu^2+^ ions.

#### 3.1.2. XRD Analysis

**Figure 3:**
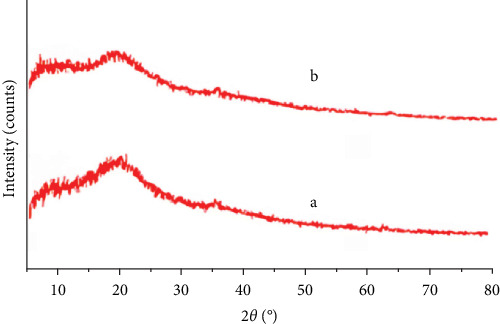
XRD patterns of the Fe_3_O_4_/chitosan–acrylic acid nanocomposite: (a) before Cu^2+^ ions adsorption and (b) after Cu^2+^ ions adsorption.

The XRD pattern of the magnetic chitosan hydrogel adsorbent reveals only two significant peaks at 8° and 21°, indicating the amorphous nature of acrylamide. A peak at 2*θ* = 10° confirms the presence of chitosan (Figure 3a,b) [3]. Additionally, peaks at 2*θ* = 35.5°, 57.2°, and 62.8° correspond to magnetic particles, supporting the presence of Fe_3_O_4_ [10, 11].

#### 3.1.3. SEM Analysis

**Figure 4:**
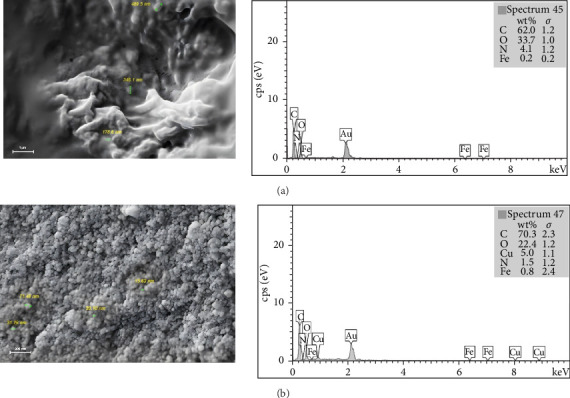
(a) The SEM image and EDS of the Fe_3_O_4_/chitosan–acrylic acid nanocomposite before the sorption of Cu^2+^ ions. (b) The SEM image and EDS of the Fe_3_O_4_/chitosan–acrylic acid nanocomposite after the sorption of Cu^2+^ ions.

The SEM findings presented in Figure 4a indicate that the magnetic chitosan hydrogel absorbent is composed of nanometer-sized particles, which can be associated with iron nanoparticles. After Cu^2+^ ions adsorption by the magnetic chitosan hydrogel, noticeable changes in the morphology of the adsorbent can be observed, as shown in Figure 4b. EDS analysis showed the adsorbent components in Figure 4a, and also the successful adsorption of Cu^2^+ ions by Fe3O4/chitosan–acrylic acid nanocomposite is confirmed as shown in the EDS analysis results in Figure 4b.

#### 3.1.4. TGA and DTG Analysis

**Figure 5:**
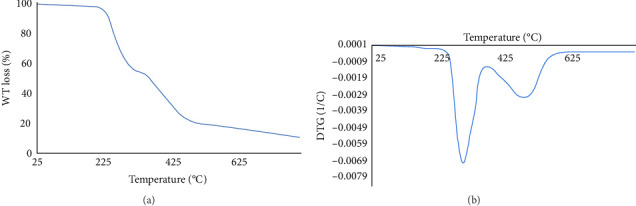
(a) TGA and (b) DTG curves of the Fe_3_O_4_/chitosan–acrylic acid nanocomposite.

The curves of the Fe_3_O_4_/chitosan–acrylic acid nanocomposite (Figure 5) indicate that the initial stage of weight loss (5%) in the magnetic hydrogel structure occurs at temperatures below 200°C, which is due to the evaporation of any remaining moisture within the hydrogel. It was observed that weight loss (35%) occurs between 225 and 360°C, attributed to the decomposition of acrylic acid and acrylamide. Further weight loss (40%) occurs around 450°C, resulting from the breakdown of chitosan [36].

#### 3.1.5. Magnetic Field Analysis

**Figure 6:**
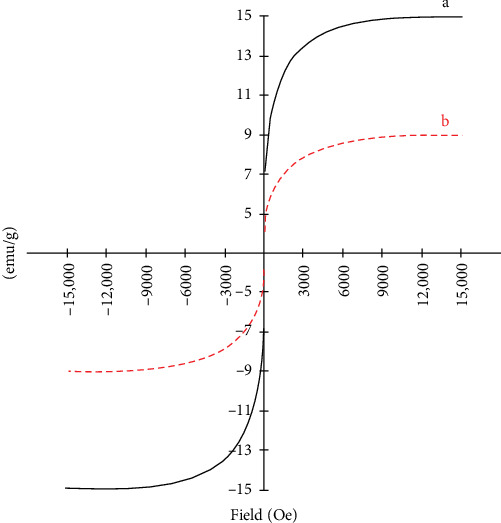
The VSM magnetization curves of Fe_3_O_4_ nanoparticles (a) and (b) the Fe_3_O_4_/chitosan–acrylic acid nanocomposite.

The Fe_3_O_4_/chitosan–acrylic acid nanocomposite was subjected to a magnetic field. The magnetic properties of the Fe_3_O_4_ nanoparticles were measured at 15 emu/g, while the magnetic chitosan hydrogel adsorbent exhibited a value of 9 emu/g. This decrease from 15 to 9 emu/g occurred due to interactions between the Fe_3_O_4_ particles and the functional groups of the magnetic hydrogel. Furthermore, the lower saturation magnetization of the Fe_3_O_4_/chitosan–acrylic acid nanocomposite likely results from the significant incorporation of Fe_3_O_4_ nanoparticles into the chitosan–acrylic acid matrix, which increases the overall mass due to the thick polymer coating on the Fe_3_O_4_ nanoparticles (Figure 6) [37].

### 3.2. BET Surface Area and Porosity Characteristics of Fe_3_O_4_/Chitosan–Acrylic Acid Nanocomposite

The average specific surface area of the Fe_3_O_4_/chitosan–acrylic acid nanocomposite was found to be 68.3 m^2^/g. The specific surface area (SBET), total pore volume (VBJH), and mean pore diameter (MPD) of the Fe_3_O_4_/chitosan–acrylic acid nanocomposite were evaluated through nitrogen (*N*_2_) adsorption–desorption analysis, as shown in Table 1. The increase in surface area after multiple uses could be due to rearrangement of the chitosan and acrylic acid polymer chains due to cyclic mechanical stresses, as well as the removal of network weaknesses (unstable crosslinks) during recovery.

### 3.3. Factors Affecting the Adsorption of Cu^2+^ Ions Onto the Biocompatible Nanoadsorbent

#### 3.3.1. pH Effect

**Figure 7:**
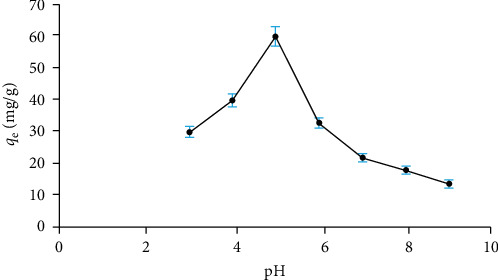
Impact of pH on the capacity of the Fe_3_O_4_/chitosan–acrylic acid nanocomposite.

To examine the effect of pH on the adsorption of Cu^2+^ ions using the Fe_3_O_4_/chitosan–acrylic acid nanocomposite, the pH of the sample solutions was adjusted within the range of 3–9. The results, as illustrated in Figure 7, indicate that adsorption capacity increased with rising pH levels up to pH 5, after which it decreases. The decline in capacity at pH levels above 5 is likely due to the formation of Cu(OH)_2_ precipitates at higher pH from Cu^2+^ ions. Conversely, the increased capacity of the Fe_3_O_4_/chitosan–acrylic acid nanocomposite at pH 5 may be attributed to the protonation of the nanoadsorbent [20, 21].

#### 3.3.2. Effect of the Amount of Adsorbent

**Figure 8:**
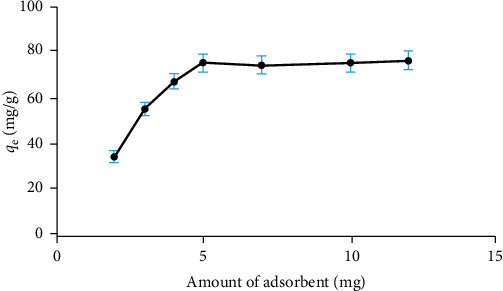
Effect of adsorbent amount on the adsorption capacity of copper ions of the Fe_3_O_4_/chitosan–acrylic acid nanocomposite.

After establishing the optimal pH of 5.0 and the ideal contact time, we investigated the effect of the amount of adsorbent on the adsorption of Cu^2+^ ions (see Figure 8). Increasing the amount of the Fe_3_O_4_/chitosan–acrylic acid nanocomposite to 5.0 mg enhanced its adsorption capacity. This improvement is attributed to the increased availability of active sites on the adsorbent. However, as we continued to increase the amount, the slope of the curve changed only slightly beyond this point. Consequently, we determined that 5.0 mg of the Fe_3_O_4_/chitosan–acrylic acid nanocomposite is the optimal amount for adsorption [32].

### 3.4. Analysis of Experimental Design Cu^2+^ Ions

The optimal conditions for the efficiency of Cu^2+^ ions adsorption were analyzed through 20 experimental designs utilizing the central composite design (CCD) method. These conditions included temperature, contact time, and the volume of a 0.010 M nitric acid eluent solvent (see Table 2). The adsorption efficiency varied from 2.0% to 86.3%. At a temperature of 26°C, the highest adsorption percentage achieved was 86.3%. The optimal contact time recorded was 14.7 min, and the optimal volume of 0.010 M nitric acid used was 8.0 mL.

### 3.5. Analysis of Variance (ANOVA)

We conducted an ANOVA based on the validation model of the CCD to evaluate the interactions among the variables. The CCD is an efficient experimental method for modeling quadratic relationships between factors and responses. It combines factorial points (for linear/interaction effects), axial points (for curvature), and center points (for error estimation), making it ideal for optimization in engineering and science. Additionally, we analyzed the ANOVA results for the interaction variables, as shown in Table 3. The optimal coefficients were derived from the ANOVA table using Equation (2) [38].

(2) $$\begin{array}{c} \text{R}=+85.26+10.68\text{A}+7.80\text{B}+11.77\text{C}-0.3750\text{AB}+4.12\text{AC}-0.1250\text{BC}-23.64{\text{ A}}^{2}-10.04{\text{ B}}^{2}-14.19{\text{ C}}^{2} \end{array}$$

**Figure 9:**
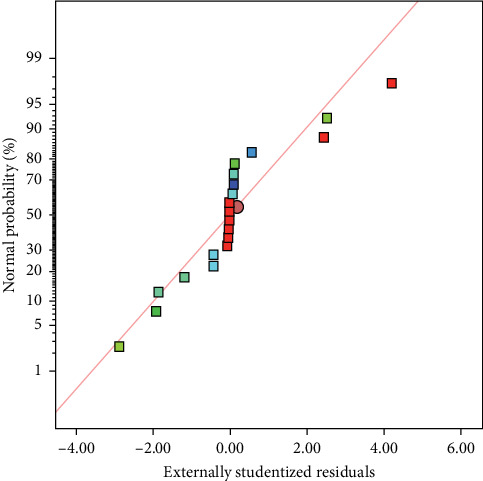
Normal plot of residuals for Cu^2+^ recovery.

An *F*-value of 10.03 indicates that the model is significant, as there is only a 0.06% chance that such a high *F*-value could occur due to random noise. *p*-values less than 0.0500 suggest that the model terms A, *B*, C, *A*^2^, and *C*^2^ are significant. Conversely, values greater than 0.1000 indicate that certain model terms are not significant. If there are too many trivial model terms—aside from those necessary to maintain the hierarchy—reducing the model may improve its performance. In this section of the fit, the lack of fit *p*-value is <0.060, indicating that the fit is not significant compared to the overall error. The extreme lack of fit *F*-value is likely due to negligible pure error, not necessarily a poor model. With a probability of 6.0%, this discrepancy is likely the result of random noise (Figure 9).

In Figure 9, the random distribution of the residuals around the zero axis indicates that the variance of the original observations is consistent for all values of Y. This suggests that the figure is satisfactory. Therefore, we can conclude that the empirical model presented is suitable for describing the adsorption efficiency based on the response subpeaks.

### 3.6. Three-Dimensional Response Surface Plots

**Figure 10:**
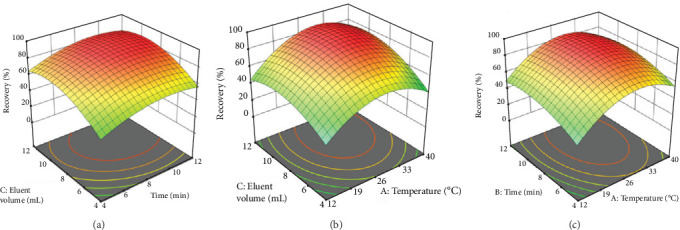
Response model for the recovery percentage of Cu^2+^ ions: (a) time and volume of 0.010 M nitric acid elution, (b) temperature and volume of 0.010 M nitric acid elution, and (c) temperature and time.

Figure 10a illustrates the relative impact of time and eluent volume on recovery. A more influence along an axis indicates that the corresponding variable has a greater influence on recovery outcomes. Additionally, the curvature of the surface around the peak provides insight into how sensitive the process is to changes in these variables. A smoother surface suggests that the process is less sensitive to small changes. Figure 10b,c highlights the relative effects of eluent volume and adsorbent temperature on recovery percentage. As before, a more influence along an axis signifies that the corresponding variable has a more significant effect on the recovery response. The curvature around the peak indicates the sensitivity of the process to changes in these two variables. Increasing the eluent solvent volume and the duration of adsorbent exposure enhances adsorption efficiency. As can be seen, the eluent solvent volume played an effective role in extracting copper ions from the adsorbent.

The CCD optimization approach was used as a flexible tool to investigate and improve the key parameters. Through the CCD method, optimal conditions were established, which included a volume of 0.010 M nitric acid, elution of 8.41 mL, an ultrasonic treatment time of 8.0 min, and a temperature of 27.42°C.

### 3.7. Effect of Competitive Ions

In the purification of natural waters, the presence of certain dissolved cations can influence the efficiency of adsorbents. This occurs because these ions may compete for adsorption on the active sites of the adsorbent's surface. Therefore, it is crucial to investigate the competition among ions when removing pollutants from aqueous solutions. The influence of these competitive ions has been studied, and as shown in Table 4, there were no significant effects on the affinities of the Fe_3_O_4_/chitosan–acrylic acid nanocomposite for the adsorption of copper ions [20].

### 3.8. Evaluation of the Reusability of the Biocompatible Nanoadsorbent

**Figure 11:**
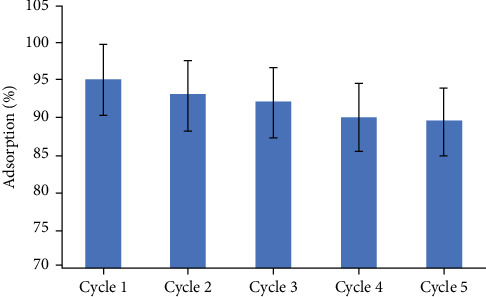
Evaluation of the reusability of the Fe_3_O_4_/chitosan–acrylic acid nanocomposite.

The reusability of an adsorbent directly impacts the economic efficiency of the adsorption system. Therefore, we studied the reusability of the biocompatible nanoadsorbent. In this study, after completing the desorption step of copper ions, the adsorbent was dried and used in the next cycle. The results, as shown in Figure 11, indicate that the adsorption efficiency remains at ~88.9% after five recycling cycles of the adsorbent. This suggests that the adsorbent can be reused up to five times without a significant reduction in its adsorption efficiency.

### 3.9. Suggested Mechanism

In this section, a hypothesis is proposed about the mechanism of copper ions adsorption by the Fe_3_O_4_/chitosan–acrylic acid nanocomposite. The synthesized adsorbent contains various functional groups that play a significant role in the adsorption of Cu^2+^ ions. The electrostatic attraction between the charged organic chemical groups of chitosan and the Cu^2+^ ions is crucial to the adsorption mechanism. Additionally, it can be said that the process of covalent bond complexation occurs between the amino groups of the polymer chains and the Cu^2+^ ions [30].

### 3.10. Adsorption Isotherms

Equilibrium properties of the Fe_3_O_4_/chitosan–acrylic acid nanocomposite were studied to fit the Langmuir (3), Freundlich (5), and Temkin (6) models. The linear forms of these models, as presented in Equations (3)–(6), are shown below:

(3) $$\begin{array}{c} C_{e}/q_{e}=\left(1/K_{L}q_{\max}\right)+\left(C_{e}/q_{\max}\right), \end{array}$$

(4) $$\begin{array}{c} R_{L=1/\left(1+K_{L}\left\|C_{0}\right.\right)}, \end{array}$$

(5) $$\begin{array}{c} \ln q_{e}=\ln K_{\text{F}}+1/n\left(\ln C_{\text{e}}\right), \end{array}$$

(6) $$\begin{array}{c} q_{\text{e}}=\left(\text{Bln}K_{\text{T}}\right)+\left(\text{Bln}C_{\text{e}}\right), \end{array}$$

where *C*_e_ (mg/L) represents the equilibrium concentration of Cu^2+^ ions. The variables *q*_e_ (mg/g) and *q*_m_ (mg/g) denote the capacity of equilibrium adsorption and the capacity of Langmuir monolayer saturation, respectively. The constants include *K*_L_ (L/mg), which is the Langmuir constant; *K*_F_ and *n*, which are Freundlich constants; *B*, representing the intensity of adsorption; *K*_T_, indicating adsorption heat; and the maximum binding energy.

The Langmuir hypothesis is based on the principle that monolayer adsorption occurs on homogeneous, free active sites. The separation coefficient (*R*_L_) is a dimensionless constant that indicates the feasibility of the adsorption process. In contrast, the Freundlich model interprets the enthalpy (Δ*H*°) of adsorption on an adsorbent with an inhomogeneous surface, suggesting that the binding tendency decreases as the degree of adsorption increases. Lastly, the Temkin model posits that the heat of adsorption decreases linearly with an increase in surface coverage and assumes a uniform distribution of binding energies across the adsorbent surface [30].

The results presented in Table 5 indicate that the Langmuir model offers a better fit of the experimental data, as evidenced by the correlation coefficient (*R*^2^). This suggests that the adsorption of Cu^2+^ ions on the Fe_3_O_4_/chitosan–acrylic acid nanocomposite occurs in a single layer and that the process is chemical in nature, taking place on a uniform surface. Additionally, the experimental results fall within the range of 0 < RL < 1, which indicates that the adsorption of Cu^2+^ ions on the Fe_3_O_4_/chitosan–acrylic acid nanocomposite is both favorable and optimal. A value of 0.20 for the RL coefficient indicates the favorable adsorption of copper ions by the Fe_3_O_4_/chitosan–acrylic acid nanocomposite [23].

### 3.11. Kinetic Studies

**Figure 12:**
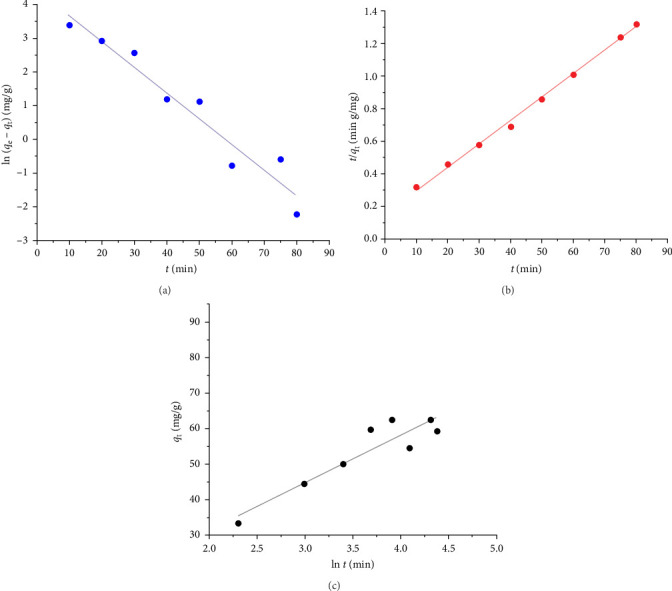
The kinetics model of (a) pseudo-first-order model, (b) pseudo-second-order model, and (c) Roginsky–Zeldovich model for Cu^2+^ ions adsorption.

The adsorption behavior of the Fe_3_O_4_/chitosan–acrylic acid nanocomposite for Cu^2+^ ions was analyzed using pseudo-first-order, pseudo-second-order, and Roginsky–Zeldovich models (see Figure 12a,b,c). The linear forms of the pseudo-first-order and pseudo-second-order equations, as well as the Roginsky–Zeldovich model, are presented in Equations (7) – (9).

(7) $$\begin{array}{c} \ln\left(q_{\text{e}}-q_{\text{t}}\right)=\ln q_{\text{e}}-K_{1}\times t, \end{array}$$

(8) $$\begin{array}{c} t/q_{t}=\left(1/K_{2}q_{e}^{2}\right)+\left(t/q_{e}\right), \end{array}$$

(9) $$\begin{array}{c} q_{t}=\left(\ln\left(\alpha \times \beta \right)/\beta \right)+\left(\text{lnt}/\beta \right), \end{array}$$

where the terms *q*_e_ and *q*_t_ (mg/g) represent the equilibrium adsorption value and the time (in min), respectively. The constants K_1_ (min^−1^), K_2_ (g(mg min)^−1^), α (mg(g min)^−1^), and *β* (g mg^−1^) correspond to the pseudo-first-order rate constant, pseudo-second-order rate constant, the rate of initial sorption according to Roginsky–Zeldovich, and the desorption constant, respectively [21]. Based on the results presented in Table 6 and Figure 12, the estimated parameters fit better with the pseudo-second-order model. This indicates that chemisorption is the rate-limiting step for the adsorption of Cu^2+^ ions onto the Fe_3_O_4_/chitosan–acrylic acid nanocomposite [21, 39].

### 3.12. Thermodynamic Properties for the Adsorption of Cu^2+^ Ions in the Fe_3_O_4_/Chitosan–Acrylic Acid Nanocomposite

This section discusses the thermodynamic parameters Gibbs free energy (Δ*G*°), Δ*H*°, and entropy (Δ*S*°) that characterize the adsorption behavior of Cu^2+^ ions on the Fe_3_O_4_/chitosan–acrylic acid nanocomposite. The negative values of Δ*G*° indicate that the adsorption process is spontaneous, and as temperature rises it becomes less negative, indicating reduced spontaneity at higher temperatures. The decrease in randomness at the solid-solution interface of the Fe_3_O_4_/chitosan–acrylic acid nanocomposite is supported by the negative values of Δ*S*°. Furthermore, the negative Δ*H*° value of −24.45 kJ/mol suggests that both physical and chemical adsorption occur during the adsorption process (see Table 7) [22].

### 3.13. Precision and Repeatability of the Method

The precision of the proposed method was evaluated using the relative standard deviation (RSD), based on measurements of Cu^2+^ ion adsorption both intraday and interday. To conduct this evaluation, five standard solutions of Cu^2+^ ions were prepared under optimal conditions, resulting in maximum adsorption of Cu^2+^ ions. The RSD values obtained were 2.45% for intraday measurements and 0.69% for interday measurements (*n* = 3). The method showed good repeatability, with an RSD of 2.10% across replicate trials.

### 3.14. Validation of the Method

The results and analytical performance are summarized in Table 8 for the proposed method under the optimized conditions. The coefficient of determination (*R*^2^) for all tested metal ions was greater than 0.9985. The limit of detection (LOD) was calculated according to the equation shown below:

(10) $$\begin{array}{c} \text{LOD}=\left(3\times \text{ SD}\right)/\text{ m}, \end{array}$$

The LOD was calculated using the formula, where SD is the standard deviation of blank signals and *m* is the slope of the calibration curve [23]. The limits of quantification (LOQ) and linearity were also determined. Both intraday and interday RSDs indicated good precision. Preconcentration factors (PFs) were calculated using the following equation [20]:

(11) $$\begin{array}{c} \text{PF}=C_{1}/C_{0}, \end{array}$$

where PF calculation uses *C*_1_, representing the analyte concentration in the solid-phase, and *C*_0_, the initial analyte concentration. Additionally, the extraction percentage (*E*%) was determined and reported for all metal ions. The LOD and LOQ values for Cu^2+^ ions were 0.15 and 10 μg/L, respectively.

### 3.15. Application to Real Samples

**Figure 13:**
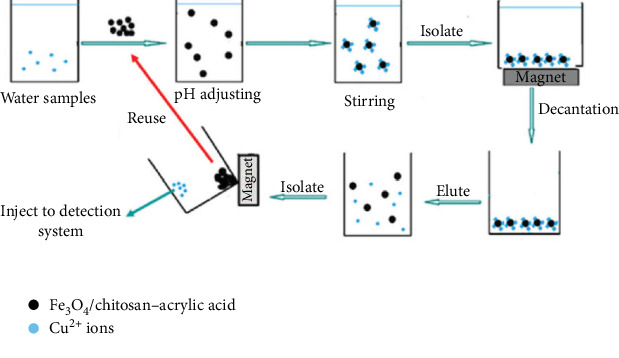
The specially designed magnetic nanobiosorbent, composed of Fe_3_O_4_, chitosan, and acrylic acid, facilitates the separation and determination of Cu^2+^ ions by combining dispersive solid-phase extraction and flame atomic absorption spectrometry.

Tap water, well water, spring water, and river water from Tehran (collected in Tehran on June 25, 2024, after running the tap for 10 min), well water (taken in Tehran on April 11, 2024), and industrial wastewater (collected in Charmshahr on February 12, 2025) were selected as the real samples for the application study of the proposed method (Figure 13). These samples stored in clean glass bottles. The optimal conditions included a sample volume of 100 mL, a pH of 5, a biocompatible nanosorbent weight of 400 mg/L, and a contact time of 14.7 min, applied for the adsorption of Cu^2+^ ions using the solid-phase extraction method. Water samples were collected in clean glass bottles and then filtered to remove colloidal and suspended particles. The results presented in Table 9 showed that the adsorption efficiencies ranged from 97% to 105.5%, with a RSD of approximately 2%. Therefore, the effectiveness of the Fe_3_O_4_/chitosan–acrylic acid nanocomposite for the adsorption of Cu^2+^ ions in real samples can be confirmed.

### 3.16. Paired *t*-Test

The results of the determination of Cu^2+^ ions using flame atomic absorption spectroscopy (F-AAS) and inductively coupled plasma (ICP) spectroscopy were analyzed with a paired *t*-test, and the findings are presented in Table 9. The results indicate that there is no significant difference between these two methods. Therefore, both techniques can be effectively used to quantify Cu^2+^ ions in water samples.

### 3.17. A Comparison of This Technique With an Alternative Method

This technique is superior to other methods due to several key advantages. It allows for a high loading capacity of Cu^2+^ ions with 30.68 mg/g and the LOD value 0.15 μg/L, operates effectively at near-neutral pH levels, and has a 15 min equilibrium time. Additionally, the Fe_3_O_4_/chitosan–acrylic acid nanocomposite can be easily separated using a magnet, eliminating the need for centrifugation or filtration. Furthermore, this method reduces the use of expensive and hazardous organic solvents when extracting from aqueous samples. But limitations such as possible interferences can also be stated for this adsorbent (see Table 10) [32–34].

## 4. Conclusion

This study employed the Fe_3_O_4_/chitosan–acrylic acid nanocomposite to effectively remove Cu^2+^ ions from water samples and enhance the properties for extracting and adsorbing Cu^2+^ ions. The structure of the Fe_3_O_4_/chitosan–acrylic acid nanocomposite was analyzed, and various amounts of the adsorbent were tested for their efficiency in extracting Cu^2+^ ions from different water samples. The maximum adsorption capacity (*q*_max_) for Cu^2+^ ions was found to be 30.68 mg/g at pH 5. The adsorption isotherms indicated that the Langmuir model best described the sorption of Cu^2+^ ions by the Fe_3_O_4_/chitosan–acrylic acid nanocomposite. Additionally, the pseudo-second-order model, with an *R*^2^ value of 0.9985, was found to be more suitable for explaining the Cu^2+^ ions sorption onto the adsorbent. In conclusion, this approach offers an effective method for measuring trace levels of copper ions in various water samples. The data show good agreement between ICP-MS and F-AAS for water samples, indicating both methods are reliable for Cu^2+^ analysis in these matrices. Key advantages include high efficiency, rapid separation rates, significant adsorption capacity, and good recovery. The Fe_3_O_4_/chitosan–acrylic acid nanocomposite can be reused up to five additional times, demonstrating its effectiveness in removing Cu^2+^ ions from aqueous solutions.

## Figures and Tables

**Table 1:** Textural properties from BET analysis.

Adsorbent	*S*_BET_ (m^2^/g)	*V*_BJH_ (cm^3^/g)	MPD (nm)
Fresh	68.3	0.947	28.8
After 5 runs	72.7	0.958	30.6
After 9 runs	78.2	0.960	38.5

**Table 2:** Results of the CCD by experimental design CCD.

Run	Temperature (°C)	Time (min)	Eluent volume (mL)	Recovery (%)
1	12	4	4	15
2	40	4	4	25
3	26	8	8	85
4	26	8	1.3	22
5	40	12	4	31
6	26	8	8	85
7	26	8	14.7	86
8	26	8	8	84
9	40	4	12	44
10	26	8	8	85
11	12	12	12	30
12	12	4	12	23
13	26	8	8	84.6
14	49.5	8	8	53
15	26	1.3	8	45
16	40	12	12	55
17	26	14.7	8	86.3
18	12	12	4	28
19	2.6	8	8	2
20	26	8	8	84.7

**Table 3:** ANOVA analysis of the Cu^2+^ ions adsorption for the quadratic response model.

Source	Sum of squares	Degrees of freedom	Mean square	*F*-value	*p*-Value	Significance
Model	15042.66	9	1671.41	10.03	0.0006	Significant
A-temperature	1553.91	1	1553.91	9.32	0.0122	—
B-time	828.26	1	828.26	4.97	0.0499	—
C-eluent volume	1885.50	1	1885.50	11.31	0.0072	—
AB	1.13	1	1.13	0.0068	0.9361	—
AC	136.13	1	136.13	0.8168	0.3874	—
BC	0.1250	1	0.1250	0.0008	0.9787	—
*A* ^2^	7958.08	1	7958.08	47.75	<0.0001	—
*B* ^2^	1435.88	1	1435.88	8.62	0.0149	—
*C* ^2^	2868.82	1	2868.82	17.21	0.0020	—
Residual	1666.63	10	166.66	—	—	—
Lack of fit	1665.86	5	333.17	2168.15	<0.060	Not significant
Pure error	0.7683	5	0.1537	—	—	—
Cor total	16709.29	19	—	—	—	—

**Table 4:** The percentage of Cu^2+^ ions adsorption in the presence of competing ions.

Interfering ions	*C*_metal ions_/*C*_interfering ion_	Recovery of Cu^2+^ ions (%)	RSD(%)
Na^+^	50	98.83 ± 2.2**^a^**	2.3
	100	98.52 ± 2.1	2.2

K^+^	50	98.70 ± 2.2	2.3
	100	97.60 ± 2.3	2.4

Ca^2+^	50	98.69 ± 2.7	2.8
	100	98.56 ± 2.1	2.2

Mg^2+^	50	99.50 ± 2.7	2.8
	100	98.50 ± 2.1	2.2

^a^Values are SD based on three individual replicate analyses.

**Table 5:** The parameters of Langmuir, Freundlich, and Temkin isotherm for the adsorption of Cu^2+^ ions by the Fe_3_O_4_/chitosan–acrylic acid nanocomposite.

Isotherm model									
Langmuir				Freundlich			Temkin		
*q*_max_ (mg/g)	*K*_L_ (L/mg)	*R*_L_	*R* ^2^	*n*	*K*_F_ (L/g)	*R* ^2^	*K*_T_	*B* (L/g)	*R* ^2^
30.68	0.02	0.20	0.99	2.63	21.15	0.97	2.14	2.89	0.96

**Table 6:** Estimated parameters for the adsorption kinetics of Cu^2+^ ions on the Fe_3_O_4_/chitosan–acrylic acid nanocomposite.

Pseudo-first-order		Pseudo-second-order		Roginsky–Zeldovich	
*q*_e_ (mg/g)	84.77	*q*_e_ (mg/g)	69.93	*α* (mg/[g min])	19.28
*k*_1_ (1/min)	0.078	*k*_2_ (g/[mg/min])	0.001	*β* (g/mg)	0.075
*R* ^2^	0.94	*R* ^2^	0.99	*R* ^2^	0.92

**Table 7:** Thermodynamic parameters for the adsorption of Cu^2+^ ions on the Fe_3_O_4_/chitosan–acrylic acid nanocomposite in (30–60°C).

Temperature (Kelvin)	*K*_c_	Δ*G*° (kJ/mol)	Δ*H*° (kJ/mol)	Δ*S*° (J/mol·K)	*R* ^2^
303.15 (30°C)	5.02	−3.51	−24.45	−66.22	94.45
313.15 (40°C)	3.11	−2.73	—	—	—
323.15 (50°C)	3.03	−1.74	—	—	—
333.15 (60°C)	2.33	−0.78	—	—	—

**Table 8:** Analytical figures of merit for the determination of Cu^2+^ ions.

Metal ion	LOD (μg/L)	LOQ (μg/L)	RSD (%) (*n* = 3)^a^		EF	LDR (μg/L)	E (%)	*R* ^2^
			Intraday	Interday				
Cu(II)	0.15	10	2.45	2.10	60	10–1000	95–105	0.99

^a^Values in parentheses are % RSD based on three individual replicate analyses.

**Table 9:** Determination of Cu^2+^ ions in real samples.

Samples	Added Cu^2+^ (µg/L)	Removal (%)	F-AAS (µg/L)	ICP method (µg/L)	*t*_exp_
Tap water	100	97	113.4 (2.2)^b^	113.6 (2.2)	1.34
Well water	100	108	128.3 (2.1)	124.5 (2.3)	1.81
River water	100	98	127.4 (2.0)	128.6 (2.0)	1.42
Spring water	100	105	113.5 (1.9)	113.1 (2.1)	1.38

^b^Values in parentheses are % RSD based on three individual replicate analyses.

**Table 10:** Comparison of the proposed method with some of the methods reported in the literature for the determination of the Cu^2+^ ions.

Metal ion	Adsorbent	LOD (μg/L)	Extraction time (min)	Recovery (%)	Reference
Cu (II)	MWCNT-Bi_2_S_3_ nanomaterial	3.98	2	92–100	[32]

Cd(II)	Magnetic allylamine	0.00056	15	98–102	—

Cu(II)	MWCNTs-TB	0.00022	15	98–102	[33]

Pb(II)	—	0.00018	15	98–102	
Cd(II)	MG-Chi/Fe_3_O_4_)	0.10	15	100	
Cu(II)	—	0.22	15	110	[34]

Pb(II)	—	0.24	15	120	
Cu(II)	Fe_3_O_4_/chitosan–acrylic acid	0.15	15	105	This work

## Data Availability

Data sharing is not applicable to this article as no datasets were generated or analyzed during the current study.
